# Epigenetic Control of Cytomegalovirus Latency and Reactivation

**DOI:** 10.3390/v5051325

**Published:** 2013-05-23

**Authors:** Xue-feng Liu, Xueqiong Wang, Shixian Yan, Zheng Zhang, Michael Abecassis, Mary Hummel

**Affiliations:** 1 Department of Surgery, Comprehensive Transplant Center, Feinberg School of Medicine, Northwestern University, 303 E. Chicago Ave., Chicago, IL 60611, USA; E-Mails: xue-liu@northwestern.edu (X.L.); xueqiong-wang@northwestern.edu (X.W.); sya016@northwestern.edu (S.Y.); zjzhang@northwestern.edu (Z.Z.); mabecass@nmh.org (M.A.); 2 Department of Microbiology-Immunology, Comprehensive Transplant Center, Feinberg School of Medicine, Northwestern University, 676 N. St. Clair St, Suite 1900, Chicago, IL 60611, USA

**Keywords:** cytomegalovirus, latency, reactivation, epigenetics, chromatin, intrinsic immunity, transplantation

## Abstract

Cytomegalovirus (CMV) gene expression is repressed in latency due to heterochromatinization of viral genomes. In murine CMV (MCMV) latently infected mice, viral genomes are bound to histones with heterochromatic modifications, to enzymes that mediate these modifications, and to adaptor proteins that may recruit co-repressor complexes. Kinetic analyses of repressor binding show that these repressors are recruited at the earliest time of infection, suggesting that latency may be the default state. Kidney transplantation leads to epigenetic reprogramming of latent viral chromatin and reactivation of immediate early gene expression. Inflammatory signaling pathways, which activate transcription factors that regulate the major immediate early promoter (MIEP), likely mediate the switch in viral chromatin.

## 1. Introduction

Human cytomegalovirus (HCMV) is an important opportunistic pathogen of the beta family of herpesviruses. Transmission of the virus occurs through exposure to infectious body fluids, including saliva, urine, breast milk, semen and blood [1]. Primary infection in immunocompetent hosts is typically subclinical, but infection of immunocompromised hosts, such as immunosuppressed recipients of solid organ or bone marrow transplants, is associated with increased risk of acute and chronic allograft rejection, infection with other opportunistic pathogens, graft failure, and death [2]. Due to the high prevalence of latent CMV, 75% of solid organ transplant patients are either newly infected with CMV, which occurs when a seronegative recipient receives an organ from a seropositive donor (designated D+/R−), or experience reactivation of latent virus present in either the donor organ or the recipient (D−/R+ or D+/R+) [3]. The highest risk of CMV disease is associated with the D+/R− combination [2,3]. Effective anti-viral drugs have reduced the incidence of post-transplant complications due to CMV infection. However, in many cases, these drugs have simply delayed the problem, and their use has been limited by toxicity and the emergence of resistant strains [4]. Immunologically immature hosts are also at risk for CMV disease. Congenital CMV infection, which occurs in utero via the placenta [5], can result in deafness, mental retardation, blindness, microencephaly, cerebral calcification, and sometimes, death [1,6]. 

Unlike alpha or gamma herpesviruses, which have a highly restricted cellular tropism, CMV infects many cell types *in vivo*, including epithelial, endothelial, smooth muscle, and connective tissue cells, as well as specialized parenchymal cells in various organs [7]. However, CMV is similar to other herpesviruses in many aspects, including virion structure, temporal regulation of gene expression, and strategies for immune evasion [8]. Like all herpesviruses, CMV has the ability to establish lifelong latent infection, in which viral DNA is present, but replicating virus is not detectable, and to reactivate from latency. HCMV latency and reactivation have been very difficult to study *in vivo*, since the virus does not infect other species. However, murine cytomegalovirus (MCMV) has proven to be a valuable model to study several aspects of CMV pathogenesis. The similarities between HCMV and MCMV of relevance here include (*i*) ability to establish latency and to reactivate [9,10,11]; (*ii*) hierarchical control of viral gene expression, in which the immediate early (*ie*) genes activate expression of the early genes, leading to viral DNA replication, late gene expression, and viral assembly; (*iii*) structure, function, and organization of immediate early genes [9,11,12], and regulation of the major immediate early promoter, which controls major *ie* gene expression [13,14]. 

## 2. CMV Latency

### 2.1. Cellular Sites of Latency

Cellular sites of HCMV and MCMV latency have recently been extensively reviewed and discussed [11]. Although many cell types support productive infection, latent HCMV infection has been documented most convincingly in cells of the myeloid lineage [10]. However, other cell types may also carry latent virus. Analysis of HCMV latency in cells within organs has been hampered by the difficulty in obtaining human tissue, by the very low frequency of latently infected cells, and the difficulty in determining whether the presence of the virus in a particular cell type is due to latent infection, or to spread of the virus after trauma-induced reactivation in deceased donors. HCMV is efficiently transmitted by solid organ transplantation, suggesting that cells within the organ harbor latent virus. While it is not possible to definitively exclude passenger leukocytes as agents of transmission, there is evidence for HCMV latency in other cell types within organs, including endothelial and epithelial cells [15,16,17]. One study sought to address the question of endothelial cell latency through analysis of saphenous vein endothelial cells taken from patients undergoing cardiovascular surgery, and concluded that these cells were unlikely to be a major site of latency [18]. However, recent studies underscore the importance of tissue-specific endothelial cell variability in the outcome of herpesvirus infection [19]. The site(s) of HCMV latency is a controversial area in need of further study.

MCMV establishes latency in multiple organs, where endothelial cells of the kidney, liver, and heart have convincingly been shown to be sites of carriage [11,20,21]. Although some studies support the view that macrophage/monocyte lineage cells also harbor latent virus [20,22], other studies do not [11,21,23]. Thus, as with HCMV, the question of the site(s) of MCMV latency has not been definitively settled.

A molecular basis for cell type specific CMV latency, despite promiscuous acute infection, has not been definitively established, but recent studies indicate that the decision between permissive and latent infection may be determined by the balance between activating and repressive factors that control transcription of viral genes upon initial infection, and this may differ among cell types [24]. 

### 2.2. Viral Gene Expression Is Repressed in Latency

The major immediate early genes encode transcriptional regulatory proteins, which are required for activation of early gene expression, and, therefore, for all subsequent phases of viral replication. These proteins are encoded by two alternatively spliced transcripts (called IE-1/IE-2 in HCMV and IE-1/IE-3 in MCMV) whose expression is controlled by the major immediate early promoter/enhancer region. In HCMV latently infected CD34+ hematopoietic progenitor cells, the immediate early genes, and most other genes associated with productive infection, are transcriptionally silent [10]. Two genes that may have roles in latency, UL138 and LUNA, are expressed in these cells, but these genes are also expressed in productive infection. Recent studies indicate that UL138 mediates degradation of the MRP1 drug transporter, and may impair generation of an HCMV-specific immune response through reduced migration of infected dendritic cells to draining lymph nodes [25], and that LUNA plays an important role in expression of UL138 in experimental models of latency and in reactivation from latency [26]. Neither of these proteins is thought to play a direct role in repressing viral gene expression in latency.

Expression of genes involved in productive infection is also repressed in mice latently infected with MCMV [11,14,20,27,28,29,30,31,32]. Although early studies of MCMV latency showed that transcripts from the immediate early region were sometimes detectable in organs of latently infected mice [29,30,33,34,35], subsequent studies have made it clear that the number of viral genomes greatly exceeds the number of transcripts. Thus, the vast majority of genomes are transcriptionally silent, but random, focal IE-1 gene expression, without progression to subsequent phases of viral replication, occurs in some cells in latent mice [9,11,27,31,32]. 

Recent studies have begun to elucidate the molecular mechanisms leading to transcriptional repression of viral genomes in latency [31,32]. Expression of some cellular genes is repressed through methylation of cytosines in CpG dinucleotides [36]. Studies of latent MCMV, as well as HSV, have shown that the DNA is not methylated in latently infected mice [37,38]. Rather, modifications of histones and other epigenetic factors bound directly or indirectly to the genome appear to hold the key to understanding the molecular basis of CMV latency.

### 2.3. Latent Viral Genomes Are Heterochromatinized

Neither HCMV nor MCMV genomes are associated with histones in the virion [39,40]. As with HSV, CMV DNA is thought to enter the nucleus after transport of DNA-containing capsids along microtubules and translocation of the DNA through the nuclear pore complex [41]. Within 30 min of infection in fibroblasts, HCMV DNA is associated with histones [42]. Studies of HCMV chromatin in latently infected cells have shown that the transcriptionally silent IE promoter is heterochromatinized, while the transcriptionally active LUNA promoter is euchromatic [43,44]. 

Epigenomic control of MCMV gene expression has been analyzed in kidneys of infected mice. In these cells, viral DNA also becomes associated with histones at very early times post-infection [32]. In latently infected mice, viral genomes are highly enriched in histones relative to cellular genes, suggesting that the DNA is in a highly condensed configuration, which is closed to the transcription apparatus [31,32]. In contrast to acute infection, where histones bound to viral lytic promoters have post-translational modifications consistent with active transcription, histones bound to lytic promoters have heterochromatic modifications, including de-methylated H3K4, de-acetylated H3K9, and mono- and di-methylated H3K9 in latently infected mice [31,32]. 

Studies of CMV and HSV latency indicate that alpha and beta herpesviruses share many features of chromatin regulation. The DNA is not methylated, and lytic genes are heterochromatinized, while genes expressed in latency have euchromatic histone modifications [45,46]. In contrast, gamma herpesvirus DNA is methylated in latently infected cells, and, although transcriptionally silent, much of the viral chromatin appears to be poised for reactivation in latently infected cells [46,47,48,49]. An important caveat to keep in mind, however, is that alpha and beta herpesvirus latency has been studied using *in vivo* models, while gamma herpesvirus latency is studied using transformed cell lines.

### 2.4. Transcriptional Repressors Are Recruited onto Viral Genomes in Latency

Repression of gene expression is mediated by recruitment of co-repressor complexes, composed of enzymes that catalyze repressive histone modifications, compact the chromatin and prevent nucleosome remodeling [50,51]. In latently infected mice, the MCMV MIEP is bound to several repressor proteins, including histone de-acetylases (HDACs) 2 and 3, Heterochromatin Protein 1γ (HP-1γ), Death-associated protein (Daxx), Recombination signal binding protein for immunoglobulin kappa J region (Rbpj), also known as CBF-1 and CSL, and its co-repressor, Corepressor Interacting with RBPJ, 1 (CIR), and Ying-Yang1 (YY1) [31,32]. Although the MIEP has been most extensively studied, viral promoters from early (M112) and late (M100) genes are also associated with transcriptional repressors in latent mice.

Chromatinization of HCMV genomes in cell culture models of infection has recently been reviewed [47,52]. The mechanisms by which viral genomes become chromatinized in the initial stages of infection are not understood. Deposition of canonical histones onto cellular DNA to form nucleosomes usually occurs as the DNA replicates in S phase [53]. However, HCMV and MCMV infect highly differentiated parenchymal cells *in vivo*, which are unlikely to be in the process of cell division. Daxx is a histone H3.3 chaperone, which deposits non-canonical histones onto DNA independently of replication [54]. Thus, Daxx may have a role in chromatinization of viral genomes during infection *in vivo*.

Histones bound to latent viral genomes are de-acetylated, and Class I HDACs are bound to viral promoters. Continued HDAC activity is likely required to maintain repression of viral gene expression, since HDAC inhibitors induce reactivation of latent herpesviruses, including HCMV and KSHV, in cell culture models [55,56]. HDACs do not bind directly to DNA, but rather, are recruited through co-repressor complexes containing proteins that interact either with methylated DNA or with transcription factors that bind to specific DNA sequences [57]. Thus, knowledge of the mechanisms by which HDACs are recruited to latent viral genomes is important to understanding how CMV latency is established and maintained. Previous studies indicate that latent MCMV DNA is not methylated [37], and thus, HDAC recruitment is likely mediated by transcription factors. Two proteins that could potentially play this role, YY1 and Rbpj/CBF-1, are bound to the MIEP in latently infected mice, which contains 12 and 10 potential binding sites for YY1 and Rbpj/CBF-1, respectively [32].

Rbpj/CBF-1 is the downstream effector of the Notch signaling pathway [58,59]. In unstimulated cells, Rbpj/CBF-1 binds to the consensus sequence YGTGRGAA (where Y=C or T, and R=A or G), and recruits co-repressors, including, CIR and HDACs to silence expression of Notch-responsive genes. Binding of ligands to membrane-bound Notch results in cleavage of the receptor and translocation of the Notch Intracellular Domain (NICD) to the nucleus, where it induces allosteric changes in Rbpj/CBF-1 that result in loss of co-repressors and formation of an activating complex containing Rbpj/CBF-1, NICD, and Mastermind. Although best known for its roles in development, Rbpj/ CBF-1 regulates many other processes, including innate and adaptive immunity [60,61,62] and viral latency and reactivation [63,64]. Several Rbpj/ CBF-1 binding sites in the MCMV MIEP overlap with NF-κB binding sites [11]. Thus, Rbpj/CBF-1 could have roles in MCMV latency, through recruitment of co-repressor complexes and competition with the activating transcription factor NF-κB. 

In addition, Rbpj/CBF-1 could function as a molecular switch to reactivate viral gene expression in response to activation of Notch-dependent or -independent signaling pathways. In gammaherpesvirus infection, CBF-1 recruits viral transactivators, EBNA-2 or Rta to activate expression of EBV or KSHV genes, respectively [46,49]. 

YY1 is a zinc finger DNA-binding protein that has dual roles as a repressor or an activator of gene expression, depending on its associated complexes and the intracellular milieu [65]. YY1 is a negative regulator of the HCMV MIEP and the HIV LTR in non-permissive cells [66,67]. YY1 may repress transcription through competition with activating factors for promoter binding sites, through interference with the function of transcriptional activators, or through recruitment of co-repressor complexes [65]. YY1 has been shown to interact with SAP30, a component of the Sin3A co-repressor complex, and to mediate recruitment of HDACs to promoters via SAP30 [68,69,70].

YY1 is subject to post-translational modifications, including phosphorylation and acetylation, and its activity, binding site affinity, interaction partners, and cellular localization can be modulated by changes in the cellular environment [69,71,72,73,74]. Thus, like Rbpj/CBF-1, YY1 could potentially have roles in both repression of viral gene expression in latency, and in reactivation of the virus through recruitment of different complexes in response to different signaling environments. 

In addition to the MIEP, HDACs are bound to MCMV promoters representative of early and late genes in latent mice [32]. The M100 late promoter, but not the M112 early promoter, has one YY1 binding site, which is occupied in latency. Neither of these promoters has binding sites for CBF-1. Binding of repressors to other promoters has not been studied. Thus, further studies are needed to characterize repressors bound to these regions and the mechanisms by which repressors are recruited to all regions of the genome.

### 2.5. Latency May Be the Default State in Viral Infection

Cell culture studies of HCMV and HSV-1 infection of permissive cells have shown that viral genomes rapidly associate with ND10 structures upon entry of the DNA into the nucleus [75,76]. ND10s are dynamic nuclear complexes, whose core components include the cellular proteins PML, Daxx, Sp100, ATRX, and HDACs. These complexes are thought to repress viral gene expression as part of an intrinsic host immune response [77]. HCMV, MCMV, HSV-1, and other viruses encode tegument proteins, which enter the cell upon fusion of the viral and host membranes, and immediate early proteins, which are transcribed in the first phase of infection, to counteract this repression and activate viral gene expression [40,75,76,77,78,79,80,81]. In addition, viral chromatin undergoes dynamic changes over the course of infection, such that histones bound to HCMV promoters at very early times post-infection have modifications consistent with transcriptional repression, which are replaced by euchromatic modifications as viral gene expression is activated [40].

**Figure 1:**
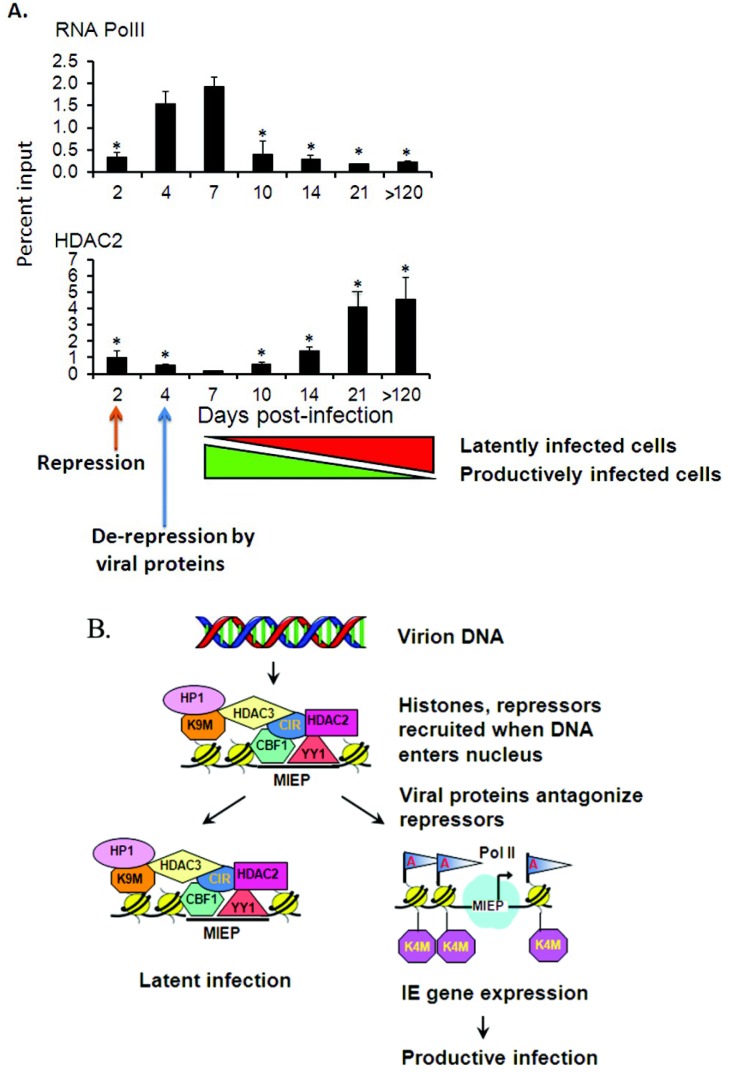
(**A**) Chromatin immunoprecipitation analysis of binding of RNA polymerase II and HDAC2 to the murine cytomegalovirus (MCMV) major immediate early promoter (MIEP) over the course of infection [32]. Results similar to those of HDAC2 were observed for binding of other repressors, including HDAC3, YY1, Rbpj, CIR, and Daxx. *, *p* < 0.05 relative to Day 7. Copyright © 2010, American Society for Microbiology; (**B**) Model for establishment of MCMV latency. Viral DNA is not complexed with histones in the virion, but is rapidly heterochromatinized upon entry in the nucleus. Co-repressor complexes may be recruited to MCMV DNA through interaction with transcription factors that bind directly to sequences in the viral genome, including YY1 and Rbpj/CBF-1. We speculate that loss of repressors in MCMV infection is due to inactivation of host cell defenses by viral proteins analogous to the HCMV pp71 tegument protein [77]. Activation of viral gene expression may fail to occur in some cells, and these cells may be the reservoir for latency. Reproduced with permission from [11].

Unlike cell culture models, the MCMV infection model permits kinetic analysis of binding of repressor proteins during all phases of infection, from acute infection, to convalescence, to latency [32]. These studies show that in kidneys of infected mice, binding of repressors, including HDACs, YY1, Rbpj/CBF-1, CIR, and Daxx, to viral genomes has three phases: binding is initially detected at the earliest phase of infection, prior to activation of viral gene expression and DNA replication, and then falls as RNA polymerase II is recruited to viral promoters and viral gene expression is activated. After a nadir at Day 7–10, when viral gene expression is most active, the percentage of viral genomes bound to repressors increases during the convalescent phase, and then plateaus (Figure 1A). 

The initial binding and subsequent loss of repressors in infected mice is consistent with host-mediated repression of MCMV gene expression through heterochromatinization of incoming viral genomes and subsequent de-repression by viral proteins, as seen in cell culture models of HCMV infection. However, even at the peak of viral gene expression, some binding of repressors to the genome was detectable [32]. These observations suggest that latency is the default state, and long term carriage of viral genomes results from failure of the virus to overcome the initial host transcriptional repression when the DNA enters the nucleus (Figure 1B). The apparent increase in MCMV genomes bound to repressors during the convalescent phase is likely due to changes in the population of cells, as productively infected cells are cleared by the host immune response, and latently infected cells harboring transcriptionally silent viral genomes predominate (Figure 1A). Eventually, all the productively infected cells are eliminated, and the percentage of genomes bound to repressors plateaus.

Live cell imaging studies have shown that ND10 proteins re-localize to viral genomes immediately upon entry of the DNA into the nucleus [82,83]. The signals that trigger recruitment of these proteins are unknown. Cellular sensors have been identified that detect the presence of pathogen-associated molecular patterns (PAMPs) localized to the cell surface, endosomes, and the cytoplasm, resulting in activation of innate immunity [84]. Entry of histone-free DNA through nuclear pores would be a highly aberrant event in an uninfected cell. It is tempting to speculate that viral DNA passing through the nuclear pore is detected as a PAMP by a protein associated with the nuclear pore complex to activate ND10s and repress viral gene expression through an intrinsic immune response. 

## 3. Reactivation of Latent CMV

### 3.1. Organ Transplantation Induces Reactivation of Viral Gene Expression

Reactivation of latent CMV is a significant infectious complication of solid organ transplantation. The highest risk of CMV disease is associated with D+/R− transplants [2,3]. Murine renal transplant models using kidneys from MCMV latently infected donor mice have therefore been developed to study the molecular mechanisms of reactivation in the context of D+/R− organ transplantation. Early studies showed that reactivation of *ie* gene expression could be induced within two days by transplanting kidneys from latently infected donor mice into naïve, allogeneic recipients [14]. Reactivation in this model correlated with expression of inflammatory cytokines, including TNF, and activation of transcription factors that bind to the MIEP, including NF-κB and AP-1. Although this model resulted in reactivation of immediate early gene expression, expression of genes representative of later stages of reactivation was not detectable. This was likely due to the very slow rate of CMV replication combined with the rapid rejection that occurs when allogeneic organs are transplanted into immunocompetent mice. Subsequent studies showed that reactivation of infectious virus in the donor kidney, and spread of the virus to recipient organs, could be induced by transplanting latently infected kidneys into immunodeficient recipient mice [85].

### 3.2. Epigenetic Reprogramming of Viral Chromatin in the *ie* Region Is Induced by Transplantation

**Figure 2:**
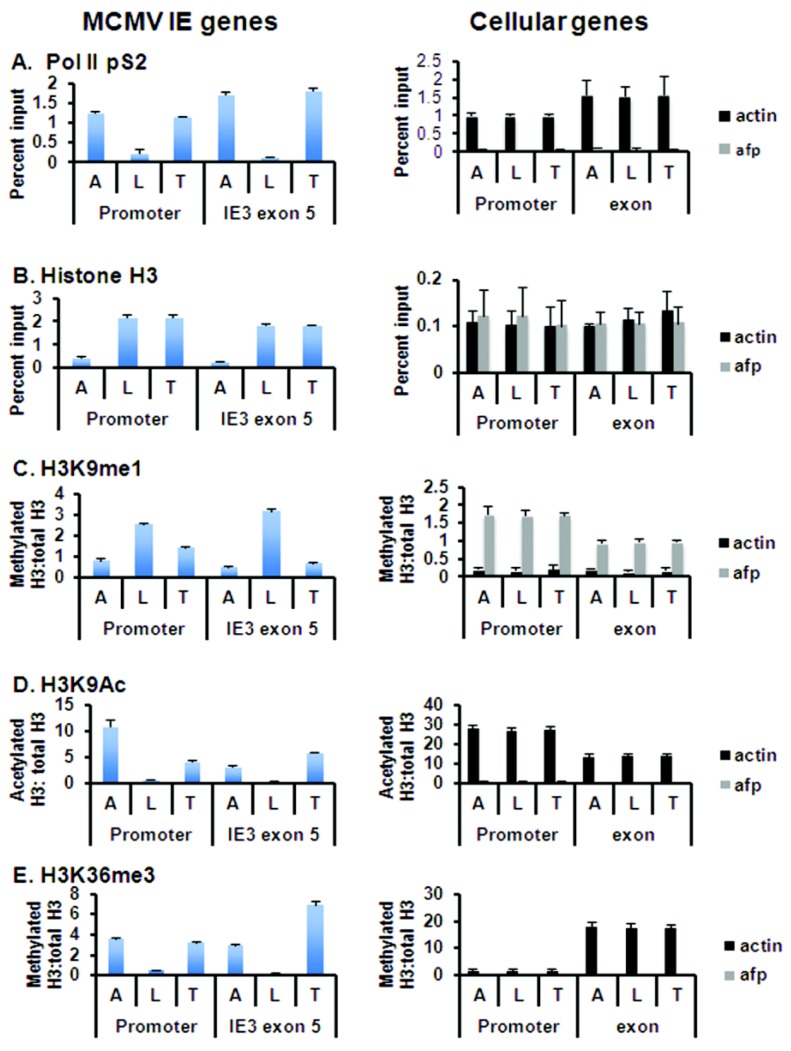
Chromatin immunoprecipitation analysis of epigenomic changes in the *ie* region induced by allogeneic transplantation. Due to the low copy number, chromatin from 30 mice was pooled for analysis of MCMV DNA in latent and transplanted kidneys. ChIP assays were analyzed in triplicate as previously described [31,32]. Results shown are the mean percent input plus standard deviation (**A** and **B**) or the ratio of modified histone to total histone after normalization to input DNA (**C**–**E**). Ratios are greater than one due to differences in antibody affinity. These results are representative of two experiments. A, acutely infected kidney; L, latently infected kidney; T, transplanted kidney; afp, alpha-fetoprotein.

Given that viral genomes are heterochromatinized in latency, reactivation of virus likely requires chromatin remodeling. We therefore analyzed changes in epigenetic factors bound to the *ie* region that correlated with transcriptional reactivation of *ie* gene expression induced by kidney transplantation. Kidneys from latently infected BALB/c mice were transplanted into wild type allogeneic C57BL/6 (B6) recipients, and the recipients were sacrificed at Day 2 (Figure 2). To determine the levels of factors bound to the MIEP in kidneys from the same mice prior to the transplant, the contralateral donor kidneys were harvested at the time of the transplant (Day 0) for analysis as latent controls. Due to the very low MCMV DNA copy number in latent mice, chromatin from 30 mice was pooled for analysis at Day 0 or at Day 2 after transplant. Acutely infected mice were analyzed in parallel as positive controls. As an additional control for comparison of different chromatin preparations, we analyzed the promoter and coding regions of two cellular genes, β-actin, a transcriptionally active gene, and α-fetoprotein, which is repressed in kidneys of adult mice (Figure 2, right panels). 

We first analyzed binding of RNA polymerase II (Pol II) to the *ie* region. Pol II can bind to promoter regions as pre-initiation complexes, and thus, binding to the promoter does not necessarily indicate active transcription [86]. However, phosphorylation of S2 of the carboxy terminal domain (Pol II pS2) is associated with transcriptional elongation, and thus, active transcription. The MCMV *ie* region encodes two differentially spliced transcripts, IE-1, containing exons 1, 2, 3, and 4, and IE-3, containing exons 1, 2, 3, and 5 [87]. We analyzed binding of Pol II pS2 to both the *ie* promoter region, and to the coding region of IE-3 (exon 5). Our results show that, as expected, high levels of Pol II pS2 were bound to the promoter and to IE-3 exon 5 during acute infection (Figure 2A, lanes A, left panel). These levels were comparable to those seen with the actively transcribed gene, β-actin. Consistent with our previous analyses, binding of Pol II to the MIEP was markedly reduced in latency (Figure 2A, lanes L, left panel). Transplantation of latently infected BALB/c kidneys into allogeneic B6 recipients induced recruitment of Pol II pS2 to both the enhancer and the IE-3 coding region at levels comparable to acute infection (Figure 2A, lanes T, left panel). Analysis of the β-actin and α-fetoprotein genes showed that differences in binding to the MCMV MIEP were not due to differences in the chromatin preparations, and that the binding was specific. These results confirmed our previous finding that transplantation of latently infected kidneys into allogeneic recipients induced transcriptional reactivation of *ie* gene expression within two days after transplant [14]. In that study, IE-1, but not IE-3 expression was detected. This discrepancy is likely due to lower abundance of IE-3 transcripts and lower sensitivity of previous assays. 

To determine whether recruitment of Pol II was accompanied by changes in viral chromatin, we analyzed changes in modifications of histones bound to the MIEP. As in our previous studies [31], we found that latent viral DNA is highly enriched in histones relative to cellular genes (Figure 2B). The percentage of viral genomes bound to H3 did not change when latent kidneys were transplanted into allogeneic recipients. The distribution of various histone modifications along a gene is typically position-dependent, with acetylation most prevalent in the enhancer region [88]. Transplantation of latently infected kidneys caused a loss of the repressive H3K9me1 mark and an increase in the activating mark H3K9Ac bound to both the promoter and coding region of the *ie* genes (Figure 2C,D). As with Pol II binding, the presence of H3K9Ac may reflect paused, rather than productive transcription [86]. However, tri-methylated H3K36 is found preferentially in coding regions, and is therefore a specific marker for elongation of transcription. Transplantation of latently infected kidneys into allogeneic recipients induced an increase in H3K36me3 in both the MIEP and IE-3 coding region (Figure 2E, left panel). No changes were observed in modifications of histones bound to cellular genes, indicating that these differences were specific to viral genes (Figure 2, right panels). These results show that transplantation of latently infected kidneys induces a switch in viral chromatin in the *ie* region, from a repressive heterochromatic state to a euchromatic configuration, leading to expression of both IE-1 and IE-3 transcripts.

### 3.3. Global Changes in Viral Chromatin Are Likely Required for Reactivation of Virus

The MCMV IE-1 and IE-3 proteins are functionally similar to HCMV IE-1 and IE-2, respectively [89]. Although there is little conservation of amino acid sequence, both HCMV and MCMV IE-1 proteins are thought to counteract host intrinsic immunity through interaction with HDACs and other ND10 components [90,91,92]. MCMV IE-3 protein shares significant homology with HCMV IE-2 in the C-terminal domain, and like HCMV IE-2, MCMV IE-3 trans-activates early gene promoters, is required to activate expression of β and γ genes during infection, and is essential for viral replication [87,93,94,95]. Thus, transcriptional reactivation of *ie* gene expression would be expected to lead to activation of transcription and remodeling of other regions of viral chromatin. Due to the difficulty in obtaining sufficient amounts of material for analysis of MCMV DNA, which is present at a copy number of ~1 MCMV genome per 10,000 cellular genomes in latent kidneys [85], only the *ie* region has been analyzed in the allogeneic kidney transplant reactivation model at Day 2. However, previous studies showed that induction of *ie* gene expression was transient in this model, with a peak of expression at Day 2, and that genes representative of later stages of replication were not detectable [14]. Rejection of transplanted organs occurs rapidly in this model, due to recognition of foreign cellular antigens, and it may therefore be necessary to use immunocompromised recipients to observe changes in other regions of viral chromatin. 

A new model for reactivation of infectious virus in genetically immunodeficient transplant recipients has recently been described [85]. As in the previous model, activation of transcription factors that bind to the *ie* promoter and reactivation of *ie* gene expression was detectable within two days after transplant (unpublished observations), but unlike the allogeneic transplant model, reactivation of infectious virus becomes detectable in immunodeficient recipients over a period of weeks. This model would therefore be useful for studying changes in viral chromatin downstream of *ie* gene expression.

### 3.4. Mechanisms of Chromatin Remodeling Induced by Transplantation

As mentioned above, YY1 and Rbpj/CBF-1 may have dual roles, both in repression of *ie* expression in latency and in activating *ie* gene expression for productive infection. However, additional activating transcription factors are likely to play a role in reactivating viral gene expression from latency through recruitment of chromatin remodeling complexes. The HCMV and MCMV *ie* enhancers are complex regions with multiple potential binding sites for transcription factors that may activate *ie* gene expression. A comparison of the potential transcription factor binding sites in the enhancers of cytomegaloviruses infecting primate and non-primate species, and a more recent analysis of the MCMV MIEP transcription factor binding sites, have been published [11,96]. Although they differ in the number and arrangement of sites, most CMV species share sites for NF-κB, AP-1, and ATF/CREB in the MIEP, and these sites may have roles in both acute infection and in reactivation from latency. The proteins that bind to these sites are dimeric complexes, which can be derived from multiple family members. Thus, there may be considerable diversity, not only in the transcription factor binding sites that are occupied under given conditions, but also in the composition and function of the complexes bound to these sites. Although there have been some genetic analyses of sites in the HCMV enhancer that regulate *ie* expression [96,97,98,99,100,101,102], there has been little analysis of transcription factors that are bound to the genome, either during productive infection or latency. Our preliminary studies indicate that the NF-κB and AP-1 sites are not occupied in kidneys of MCMV latently infected mice, but the NF-κB p65 subunit and the AP-1 junD subunit are recruited to the MIEP when latently infected kidneys are transplanted into allogeneic recipients (unpublished observations). NF-κB p65 is activated by inflammatory cytokines and PAMPs through the canonical pathway, and activates expression of many genes involved in innate and adaptive immunity [103]. JunD is activated by oxidative stress, and induces expression of genes that promote survival and protection from reactive oxygen species induced by oxidative stress [104,105,106,107]. 

**Figure 3:**
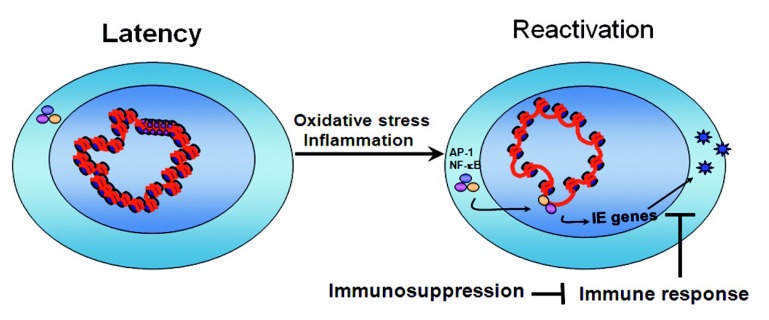
Model for reactivation of CMV from latency. In latently infected cells, episomal viral genomes are heterochromatinized, and transcription factors that drive IE gene expression, such as NF-κB and AP-1, are inactive (NF-κB depicted as cytoplasmic trimeric complex of p65, p50, and the inhibitory subunit, IκB). We propose that inflammatory cytokines and oxidative stress lead to activation of transcription factors that bind to the MIEP and induce chromatin remodeling to activate *ie* gene expression. Reactivation of *ie* gene expression does not lead to reactivation of virus in immunocompetent individuals, but in immunosuppressed individuals, reactivation of IE gene expression leads to production of infectious virus.

Allogeneic transplantation induces both oxidative stress, due to ischemia/reperfusion injury, and an inflammatory response due to allorecognition of foreign antigens. At Day 2 post-transplant, many inflammatory cytokines, antigen-presenting molecules, and co-stimulatory molecules are up-regulated in allogeneic kidney transplants [14,108]. Many of these factors are known to activate signaling pathways that lead to activation of NF-κB and AP-1, and thus, could contribute to reactivation of *ie* gene expression (Figure 3). 

CMV infection in transplant recipients is associated with infection with other pathogens, with episodes of acute rejection, and treatment with lymphocyte-depleting regimens that induce release of inflammatory cytokines [2,3]. Several investigators have therefore suggested a link between an inflammatory immune response and reactivation of CMV mediated by activation of transcription factors that bind to the MIEP to initiate viral gene expression [11,13,109,110,111]. Determining which transcription factors are required for reactivation of CMV from latency, their roles in chromatin remodeling, and the signaling pathways by which these factors are activated, are significant, but important, challenges for the future.

## 4. Conclusions

CMV infection initiates a complex battle for survival between the virus and host. Although CMV encodes a wealth of genes dedicated to subverting host innate and adaptive immune responses [112], the host is generally able to clear cells producing infectious virus in immunocompetent individuals. The end result of this battle is therefore a standoff between the virus and host, in which the virus is able to persist for the life of the host, but does not cause active disease. It is becoming increasingly clear that this latent state is established by epigenetic factors that mediate heterochromatinization of viral genomes to silence viral gene expression. The virus is able to persist indefinitely in these cells because they are not expressing viral proteins, and are therefore invisible to the host immune response. Transcriptional repression may be established at the outset of infection as a result of a third type of immunity, which has been called an intrinsic immune response [79,80], and the reservoir of latently infected cells may arise from failure of the virus to overcome this initial repression in some cells.

Although CMV wins the virus/host battle in one sense, in that it is able to survive in the face of host immunity, replication is the only successful outcome for a virus. Latency is a dead end, and survival of latent virus is dependent on survival of the host. Reactivation of the virus in response to events that threaten the survival of the host, such as infection with another pathogen, would therefore be expected to have an adaptive advantage. Previous studies have shown that reactivation of MCMV *ie* gene expression is rapidly induced by allogeneic transplantation, and by inflammatory mediators, including TNF and LPS [14,30,109]. Reactivation of HCMV can be induced by allogeneic stimulation, dendritic cell maturation and IL-6 [43,113,114,115]. These stimuli are all associated with an inflammatory immune response. Collectively, these observations lend credence to the hypothesis that CMV has evolved to escape from hosts where its survival may be compromised.

Here, we have demonstrated that reactivation of latent MCMV gene expression induced by organ transplantation occurs through epigenetic reprogramming, which results in changes in modifications of histones bound to viral DNA and recruitment of activating transcription factors. Although inflammation has been implicated in reactivation, the specific signaling pathways that mediate this response, either in immunocompetent individuals or in transplant recipients, are unknown. The key outstanding questions with respect to epigenetic control of CMV latency and reactivation are (1) how are viral genomes chromatinized in the initial stage of infection; and (2) what are the signaling pathways that lead to chromatin remodeling and transcriptional reactivation of viral gene expression? The answers to these questions may lead to new therapies to eradicate latent virus or to prevent reactivation in immunocompromised hosts.
